# Integrating palliative care into primary healthcare systems: Advocacy efforts, milestones and challenges in Asia

**DOI:** 10.51866/cm0007

**Published:** 2024-10-23

**Authors:** GVC Fernando, Teguh Kristian Perdamaian

**Affiliations:** 1 MBBS, PgDFM, MCGP, FRCGP, DipPallMed, Master (Palliative Care), Faculty of Graduate Studies, University of Sri Jayewardenepura, Sri Soratha Mawatha, Gangodawila, Nugegoda, Sri Lanka. Email: chemetf@gmail.com; 2 BMedSc, MPH, Faculty of Medicine, Universitas Kristen Duta Wacana, Yogyakarta, Indonesia.

**Keywords:** Primary healthcare, Primary care, Palliative care, Access to healthcare, Asia

## Abstract

Palliative care is a vital component of primary healthcare systems, especially in Asia, where the ageing population is expected to increase significantly in the coming years. Integrating palliative care into primary healthcare systems is a crucial strategy for achieving universal access to palliative care. It is necessary to take concurrent actions to achieve this integration, including integrating palliative care into public health policies, educating primary healthcare workers, establishing appropriate service structures and ensuring the availability of controlled medications. Healthcare professionals involved with primary care, often led by physicians, play a significant role in driving the implementation of primary palliative care in Asia, as evidenced by their involvement in community- and home-based palliative care in India and primary palliative care for patients with cancer in Indonesia. However, there are challenges associated with implementing these actions in each country. Therefore, it is crucial to examine the ongoing advocacy efforts, milestones, obstacles and strategies that shape this process in the Asian context.

## Introduction

Palliative care is a comprehensive, patient-centred approach that emphasises improving the quality of life for individuals with life-threatening illnesses and their families, addressing physical, psychosocial and spiritual suffering.^1^ By 2050, it is anticipated that approximately a fourth of populations in the Asia-Pacific and a fifth of populations in Southeast Asia will be 60 years or older, which is likely to result in a surge in age-related health issues such as cancer, organ failure, cognitive decline and multimorbidity.^2,3^ Given this trend, it is imperative to prioritise palliative care as a vital component of public healthcare systems in Asia. As of2020, only Australia, China, Japan, New Zealand, Singapore, South Korea, Taiwan and Thailand in the Western Pacific region and none of the nations in the Southeast Asian region have successfully integrated palliative care into their primary healthcare services.^4^ Thus far, different healthcare models and strategies have been implemented across countries, with varying levels of success.^5,6^

One way to ensure universal access to palliative care is its integration into primary healthcare systems.^7,8^ The principles of palliative care such as continuity of care, comprehensive care, patient-centred care and coordinated care align with those of primary care, making such integration suitable and feasible.^9^ Across many developed countries, primary palliative care is typically provided by primary care physicians, referred to as general practitioners or family physicians. However, this is not necessarily the case in most low-to-middle-income countries (LMICs) because their primary healthcare systems are immature and diverse.

Primary palliative care is defined as the practice of palliative care provided by primary healthcare workers who are the main providers of integrated healthcare for people in local communities throughout their lives (Munday et al., 2019). This approach includes identifying and initiating palliative care as part of integrated and holistic chronic disease management, collaborating with specialist palliative care services where they exist and enhancing the underlying professional capabilities in primary care. The three principal functions of primary palliative care are identifying patients who may benefit from a palliative care approach, conducting holistic assessment and planning and coordinating care.^11^ These proactive measures taken within the premise of primary palliative care have been shown to improve symptoms and quality of life among patients, minimise unnecessary hospitalisations and lower healthcare costs.^12^ Therefore, to align with the efforts to achieve universal health coverage in terms of palliative care, LMICs must strive to achieve this integration.

Integrating palliative care into primary healthcare systems continues to gain global momentum. According to^13^, four key actions must be taken concurrently to establish primary palliative care in any country or socioeconomic setting. These include integrating palliative care into public health policies, educating primary healthcare workers, establishing an appropriate service structure, ensuring the availability of medications and controlling narcotic drugs used for pain management. However, there are distinct challenges associated with implementing these actions in individual countries or regions. Considering the unique characteristics of diverse primary healthcare systems that prevail in Asia, particularly the significant role of community engagement and community-centred approaches^14,15^, it is crucial to examine the advocacy efforts, milestones, obstacles and strategies that shape this process in the Asian context.

## Promoting and advocating for primary palliative care in the Asia-Pacific region: Role of primary care physicians

As previously outlined, the primary healthcare models utilised in the Asian region are diverse, with multiple professional roles leading primary care teams across various settings, ranging from community-based centres to local primary care centres. In urban settings, primary care is generally overseen by primary care physicians holding a medical undergraduate degree or equivalent or by general practitioners and family physicians who have undergone postgraduate training in general practice or family medicine. In this context, primary care physicians play a significant role in driving the implementation of primary palliative care in the Asian region regardless of their training and qualifications.^16,17^

Although there are limited reports on the role of primary care physicians in primary palliative care in Asia, there are some examples.^18^ For instance, in some areas of India, community- and home-based palliative care is well established, with some teams led by physicians trained in primary palliative care.^19,20^ Moreover, there is growing interest among Indian family practitioners in offering primary palliative care in their practices, supported by needs assessments and educational initiatives, such as the integration of palliative care into medical curricula and continuing medical education.^21-23^ Similarly, in Indonesia, particularly in Jakarta and Surabaya, primary care physicians actively provide primary palliative care to patients with cancer referred from hospitals.^24,25^ These efforts are made possible with the support of non governmental organisations and regional hospitals that train primary care physicians and community teams.^26^

With a vision to overcome obstacles in providing palliative care in primary care settings in Asia, a special interest group dedicated to primary palliative care (PrimPallCare-SIG) was established within the Asia Pacific Hospice Palliative Care Network (APHN) in 2022.^27^ This group also operates within the Asia-Pacific and South Asian regions of the World Organization of Family Doctors (WONCA). The PrimPallCare-SIG serves as a platform for promoting advocacy, policy, education, research and development to improve the provision of palliative care in primary care settings. By conducting knowledge exchange and capacity-building initiatives, the group aims to engage in an evidence-based approach to equip healthcare professionals with the necessary skills to provide high-quality palliative care to patients in their respective communities.

Collaborating with other regions, such as Europe, Africa, Latin America and North America, presents opportunities to learn from one another, synergise advocacy efforts through joint projects and share resources. As an APHN affiliate, the PrimPallCare-SIG collaborates with several global-scale organisations on projects of international significance. These organisations include the Cancer and Palliative Care Special Interest Group and Working Party on Education of the WONCA, the advocacy arm of the International Association for Hospice and Palliative Care, leadership of the Worldwide Hospice and Palliative Care Alliance and International Children’s Palliative Care Network.

The PrimPallCare-SIG has actively participated in numerous conferences relevant to primary care and palliative care throughout Asia, Europe and other continents. During these scientific meetings, the group has introduced the emerging field of primary palliative care to healthcare professionals and other relevant parties. These conferences have facilitated the formation of valuable partnerships with like-minded individuals and organisations. One of the principal regional collaborative initiatives of the PrimPallCare-SIG is conducting a survey to assess the current state of knowledge, policy and practice related to primary palliative care in Asia. This survey also aims to better understand the concept of primary palliative care in the Asian context. Additionally, the special interest group hosts a bi-monthly webinar series to educate relevant regional primary healthcare professionals, which is open to interested members worldwide.

One of the principal collaborators of the PrimPallCare-SIG is the Cancer and Palliative Care Special Interest Group of the WONCA. Together, these two special interest groups intend to expand palliative care educational opportunities for healthcare professionals engaged in primary care provision globally. One of the key collaborative projects between these two groups initiated by the PrimPallCare-SIG is developing a core competency framework for physicians working in primary care settings. Partnerships have been formed with the regional and global organisations relevant to palliative and primary care mentioned above to accomplish this ambition. In addition, many research projects are underway, where members of the two groups are actively engaged. One project aims to assess telemedicine’s effectiveness, feasibility and acceptability in delivering primary palliative care to rural communities. Another project initiated by the Palliative Care Sub-Committee of the Lancet Commission on Human Crisis of Cancer engages with multiple stakeholders in exploring strategies to deliver palliative care to patients with cancer in LMICs in a cost-effective manner.

## Future challenges and opportunities

Although primary palliative care can potentially improve access to palliative care, it also presents challenges and opportunities for implementation. Barriers may arise from human resources, external factors and organisational factors. For example, the scarcity of primary care physicians competent in palliative care in the Asian region may require additional training and education. Implementation can also be hindered by a lack of interest among primary care physicians in applying a palliative care approach to the communities they serve. Further, internal organisational factors within the primary care team, such as funding, teamwork and administrative burden, as well as external factors such as universal health coverage and community attitudes towards palliative care can impact implementation.

To address the abovementioned issues, medical schools can develop comprehensive training programmes on basic or essential palliative care by collaborating with organisations such as but not limited to the APHN or WONCA Similarly, postgraduate training programmes must focus on educating primary care physicians about the best practices and evidence-based interventions to provide high-quality palliative care to patients with life-limiting illnesses. Additionally, continuous medical education and ongoing professional development opportunities can help these physicians stay up to date on the latest advancements and guidelines for palliative care delivery in out-patient, home and community settings.

Educational campaigns focusing on healthcare professionals and the public must be aimed at enhancing awareness of the unique palliative care approach and its benefits. These campaigns may motivate and lobby collective advocacy efforts from both groups to drive policy and practice to incorporate palliative care into primary healthcare in their respective settings as an essential human right.^28^ Additionally, as evidenced in different settings, financial incentives and support systems can motivate physicians to incorporate palliative care into their practice.^29,30^ Finally, collaborating with patient advocacy groups and community organisations can help raise awareness of the importance of palliative care and increase its adoption among primary care physicians.

Notably, there is growing interest among primary care physicians in receiving training in primary care. This trend is reflected by the formation of the Young Doctors’ Movement of the WONCA and the World Health Organization’s Primary Health Care Young Leaders’ Network.^31,32^ Aligned with this developing momentum, including primary palliative care in the primary care curricula at both undergraduate and postgraduate levels may support capacity building, especially for primary care physicians as leaders in its implementation. Integrated healthcare professional training programmes must be developed and implemented in a sociocultural, context-sensitive manner to optimise the interdisciplinary management of patients holistically.^33^

The increasing momentum towards integrating palliative care into primary care models could be leveraged to simultaneously enhance primary care systems. Eventually, these trends can expand primary palliative care models, increasing people’s accessibility to palliative care and enhancing the overall quality of primary care. A palliative care approach can help address the gaps in primary healthcare by providing a holistic, integrated and person-centred care framework, thus improving the overall quality of care and ensuring that all individuals have access to the support they need to live well until the end of their lives.
